# Laparoscopic management of paraganglioma in a pregnant woman: a case report

**DOI:** 10.1590/S1677-5538.IBJU.2017.0698

**Published:** 2018

**Authors:** Mohammad Hadi Radfar, Behnam Shakiba, Amir Afyouni, Hassan Hoshyar

**Affiliations:** 1 Urology and Nephrology Research Center, Shahid Labbafinejad Hospital, Shahid Beheshti University of Medical Sciences, Tehran, Iran

**Keywords:** Laparoscopy, Paraganglioma, Pregnant Women

## Abstract

**Introduction:**

Paraganglioma is an extremely rare catecholamine-producing tumor during pregnancy. Paraganglioma carries high risks of fetal and maternal mortality during pregnancy. We report a pregnant woman with paraganglioma in the second trimester.

**Case Description:**

A 24-year-old pregnant woman presented with severe hypertension in the 17th week of gestation. Hormonal examination and Magnetic Resonance Imaging (MRI) confirmed the diagnosis of extra adrenal pheochromocytoma (paraganglioma). She underwent laparoscopic tumor excision successfully.

**Conclusions:**

A high index of suspicion is needed to diagnose paraganglioma in a pregnant patient with hypertension. Laparoscopic tumor removal for paraganglioma seems to be a feasible and safe procedure during pregnancy.

## INTRODUCTION

Paraganglioma and pheochromocytoma are extremely rare catecholamine-producing tumors during pregnancy and have been estimated to occur in one in 54.000 pregnancies (1). Extra adrenal pheochromocytoma (paraganglioma) is less common than adrenal pheochromocytoma in pregnancy. The incidence of paraganglioma during pregnancy is about 19% of catecholamine-producing tumors (2). Paraganglioma carries high risks of fetal and maternal mortality during pregnancy. Therefore, early diagnosis and appropriate management decrease the maternal and fetal mortality. Definite treatment of paraganglioma is surgical tumor removal but before decision of surgical intervention, maternal and fetal safety should be considered (3).

We report a pregnant woman with paraganglioma which was diagnosed in the second trimester, and underwent laparoscopic tumor excision successfully.

## CASE DESCRIPTION

**Figure 1:**
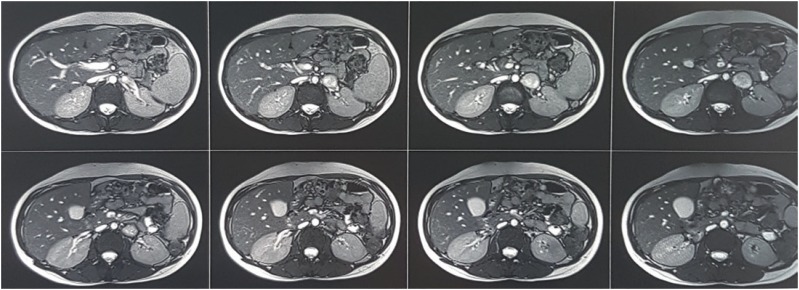
Magnetic Resonance Imaging (MRI) of abdomen showed a round soft tissue mass measuring 3×3.5cm medial to left renal hilum, compatible with extra adrenal pheochromocytoma (paraganglioma).

A 24-year-old pregnant woman presented to a local private clinic with severe intermittent headache. She was at the 17th week of her first single fetus pregnancy. On physical examination, the patient had systolic blood pressure at 220-240mmHg and diastolic blood pressure at 140mmHg. No other abnormalities were noted. With diagnosis of pregnancy-induced hypertension (PIH), antihypertensive treatment was begun and patient was referred to an obstetrics clinic. Gynecologist refused the diagnosis of PIH, because, PIH develops after 20 weeks of gestation. Past medical history revealed a history of palpitation and sweating for about 1 year and she has not undergone any medical workup. During pregnancy, her blood pressure ranged from 125/75mmHg to 145/85mmHg. Despite the maximal dose of antihypertensive treatment, BP persisted uncontrolled. Abdominal ultrasonography showed a mass measuring 31×33mm medial to left renal hilum. The results of laboratory studies, including blood cells count, blood chemistry, urine analysis, urinary albumin and blood electrolytes, were within normal limits. Hormonal examination showed markedly elevated 24-hour urinary excretion of metanephrines and normetanephrines. The other hormonal assessment including adrenocorticotropic hormone, cortisol, aldosterone and plasma renin activities were in normal range. Magnetic Resonance Imaging (MRI) of abdomen showed a round soft tissue mass measuring 3×3.5cm medial to left renal hilum, anterior to renal artery and vein (Figure-1). This tumor was compatible with extra adrenal pheochromocytoma (paraganglioma).

**Figure 2:**
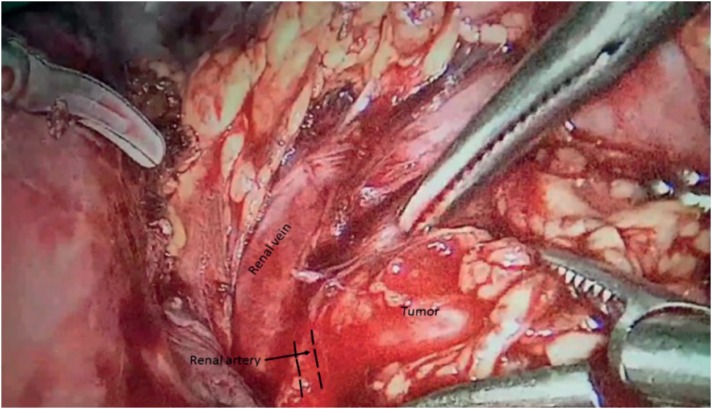
Laparoscopic transperitoneal tumor excision is shown. The tumor was adherent to the underlying renal vessels.

Alpha-adrenergic blockade with phenoxybenzamine was performed for 10 days and blood pressure was maintained under 140/90mmHg. At 19 weeks of gestation, she underwent laparoscopic tumor removal. Laparoscopy was done by the transperitoneal approach in left flank position, as it best exposes the tumor and renal vessels. We used the Hasson technique to create pneumoperitoneum and the operation was done by four working trocars. It was necessary to mobilize the colon and tail of the pancreas. The tumor was located very adjacent to the major blood vessels of the left kidney (Figure-2). The tumor was excised effectively without any renal vascular damage. The operation was uneventful and the patient blood pressure was controlled without medications. She was discharged after 5 days with a blood pressure of 140/75mmHg and a heart rate of 86 beats per minute. Microscopic histopathology revealed extra adrenal pheochromocytoma (paraganglioma). The patient had a normal vaginal delivery of a healthy baby at 39 weeks of gestation.

## DISCUSSION

During pregnancy, the prevalence of pheochromocytoma and paraganglioma is extremely rare and has been estimated about 0.007% of all pregnancies (4). These tumors derive from chromaffin cells. Paraganglioma (extra adrenal pheochromocytoma) is less common than pheochromocytoma in pregnancy, and has been reported that paraganglioma accounted for only 19%-25% of chromaffin cell tumors, whereas the majority were pheochromocytomas (2, 5). Maternal and fetal mortality in undiagnosed and untreated pheochromocytoma and paraganglioma is cited about 40-50%. In contrast, after early diagnosis and appropriate treatment, maternal and fetal mortality is decreased to 5% and 15%, respectively (1).

Although some patients with paraganglioma remain asymptomatic, some patients present with life-threatening medical problems. The typical clinical manifestations of paraganglioma are paroxysmal symptoms such as hypertension, headache, sweating, and palpitations. The most common symptom is paroxysmal or sustained hypertension. The diagnosis can be easily missed during pregnancy because some signs and symptoms of paraganglioma specially hypertension, is generally appearing to pregnancy-induced hypertension. This similarity in clinical manifestations and the rarity of the disease, leads to delayed diagnosis. Therefore, the diagnosis of about 20% of the patients are made during labor or immediately postpartum.

The most prevalent cause of hypertension in pregnant women is pregnancy induced hypertension (PIH), which develops after 20 weeks of gestational age (1, 6). In the present case, hypertension developed before 20 weeks of gestation; accordingly, complete medical history and physical examination were mandatory in the present case for diagnosis of secondary causes of hypertension.

If clinical signs and symptoms are suggestive of pheochromocytoma and paraganglioma, immediate biochemical tests should be done. Pregnancy does not change plasma or urine level of metanephrines (normetanephrine and metanephrine). Measurement of metanephrines, either in blood or in urine, is the preferred test to rule out or to confirm the diagnosis of pheochromocytoma and paraganglioma (7).

After clinical and biochemical diagnosis of paraganglioma, the best imaging modality for localization of the tumor in pregnant women is the magnetic resonance imaging (MRI). Metaiodobenzylguanidine (MIBG) scan is contraindicated during pregnancy period (8).

Due to the rarity of paraganglioma during pregnancy, existing recommendations for the management are based on case reports and expert opinion. Based on these evidences, the ideal management is directly related to gestational age. When diagnosis is set before the 24th week of gestation, the best treatment is surgical tumor removal in the second trimester. Laparoscopic tumor removal is the preferred surgical approach (2). In these patients, after successful tumor removal, cesarean section and vaginal labor are equally safe. When the tumor is diagnosed after 24 weeks of gestation, surgical tumor removal should be delayed after delivery either immediately or at later dates. These patients should be treated with antihypertensive drugs until delivery and surgical tumor excision. In these patients, cesarean section is the preferred mode of delivery, because vaginal labor leads to higher fetal and maternal mortality than cesarean section (1).

Pregnant and non-pregnant patients with pheochromocytoma or paraganglioma should undergo appropriate preoperative catecholamine blockade. Alpha-adrenergic blockade should be started as soon as possible after diagnosis. In both pregnant and non-pregnant patients, phenoxybenzamine is the alpha blocker of choice. An alternative drug for alpha blockage is prazosin. Establishment of appropriate α-adrenergic blockade generally requires 10 to 14 days of treatment. The target blood pressure in pregnant patients is controversial because very low blood pressure can compromise the uteroplacental circulation and impair fetal growth. Tachycardia or arrhythmia can be treated with beta-adrenergic blockers only after some days of appropriate alpha adrenergic blockade (3, 6).

In conclusion, early diagnosis and proper management of paraganglioma significantly decrease the rate of fetal and maternal mortality. Despite all diagnostic and therapeutic improvement over the last decades, a high index of suspicion is needed to diagnose paraganglioma in a pregnant patient with hypertension. Laparoscopic tumor removal for paraganglioma seems to be a feasible and safe procedure during pregnancy.
